# Involucrasin B Inhibits the Proliferation of Caco-2 Cells by Regulating the TGFβ/SMAD2-3-4 Pathway

**DOI:** 10.3390/molecules29030686

**Published:** 2024-02-01

**Authors:** Zi Wang, Wanjun Lin, Meina Shi, Yu Hou, Jiachen Liu, Zifeng Huang, Xuening Zhang, Yanchao Yang, Beijia Liu, Zhuya Yang, Wenzhe Ma

**Affiliations:** 1 State Key Laboratory of Quality Research in Chinese Medicine, Macau University of Science and Technology, Macau 999078, China; 2009853ucw30009@student.must.edu.mo (Z.W.); wjlin@must.edu.mo (W.L.); shimnluck@163.com (M.S.); houyu2019swmu@163.com (Y.H.); 2109853dct20004@student.must.edu.mo (J.L.); zifenghuang2021@163.com (Z.H.); zhangxn56@mail3.sysu.edu.cn (X.Z.); 2220023685@student.must.edu.mo (Y.Y.); 2220023731@student.must.edu.mo (B.L.); 2 School of Traditional Chinese Medicine, Yunnan University of Traditional Chinese Medicine, Kunming 650500, China

**Keywords:** involucrasin B, *Shuteria involucrata* (Wall.) Wight & Arn, colorectal cancer, TGFβ RII, SMAD3, CDK, cell cycle

## Abstract

(1) Background: Colorectal cancer (CRC) is the third most common malignant tumor worldwide and the second most common cause of cancer death. However, effective anti-CRC drugs are still lacking in clinical settings. This article investigated the anti-proliferative effect of involucrasin B on CRC Caco-2 cells. (2) Methods: This study employed a sulforhodamine B (SRB) method, colony formation experiments, flow cytometry, FastFUCCI assay, dual luciferase assay, and Western blot analysis for the investigation. (3) Results: The SRB method and colony formation experiments showed that involucrasin B exhibited an inhibitory effect on the Caco-2 cells cultured in vitro. Subsequently, the flow cytometry, FastFUCCI assay, and Western blotting results showed that involucrasin B induced cell cycle arrest in the G1 phase dose-dependently. Involucrasin B significantly enhanced the TGFβ RII protein level and SMAD3 phosphorylation, thus inhibiting the expression of CDK4 and cyclin D1 and causing G1 cell cycle arrest. (4) Conclusion: This study shows that involucrasin B exerts its anti-proliferative effect by regulating the TGFβ/SMAD2-3-4 pathway to cause G1 cycle arrest in Caco-2 cells.

## 1. Introduction

Colorectal cancer (CRC), a prevalent digestive system cancer, ranks as the third most common malignant tumor globally, comprising about 10% of all cancers, and is the second leading cause of cancer-related deaths [1,2]. CRC’s incidence and mortality rates are on the rise in most countries, especially in the United States, Europe, and Asia [3,4]. Siegel et al. estimated that around 153,020 new cases of CRC would be reported in the United States in 2023 [5]. Presently, the standard clinical management for CRC involves radical surgery combined with adjuvant chemotherapy, which has proven effective in improving its early prognosis [6]. However, despite treatment advancements, advanced CRC still exhibits high mortality rates [7]. Studies suggest that while screening has reduced early morbidity, there has been an increase in the occurrence and fatality of advanced CRC in recent years [6,8]. The chemotherapy limitations in current CRC management include systemic toxicity, low tumor specificity, and acquired drug resistance [8]. Therefore, it is crucial to introduce new compounds that are highly efficient, safe, stable, and effective for the clinical management of CRC.

About 80% of approved anticancer drugs originate from natural compounds or their derivatives, highlighting natural products as the primary source of new antineoplastic medications. Flavonoids, characterized by a 2-phenyl chromone structure, offer various biological benefits, such as cardiovascular protection and antibacterial, antiviral, anti-tumor, antioxidant, anti-inflammatory, antihypertensive, hypolipidemic, immune-boosting, antitussive, and spasmolytic activities [8,9]. Numerous flavonoids have exhibited anti-tumor activities on CRC cells [10,11,12]. We previously isolated, characterized, and studied the anti-tumor properties of two rare 5-dehydroxy/5-demethoxy 2′,3′,4′-trisubstituted flavanones, involucrasin A and B, derived from *Shuteria involucrata* [13]. Involucrasin A (C_21_H_22_O_5_) was a white amorphous powder that was soluble in dimethyl sulfoxide. Wei et al. evaluated the anticancer activity of involucrasin A in HCT-116 CRC cells. Their findings revealed that involucrasin A primarily exerts its anti-tumor effects by modulating the Akt/MDM2/P53 pathway [8]. While the anti-tumor activities and mechanism of action of involucrasin A on CRC cells have been recently investigated, the impact of involucrasin B remains unclear.

CRC cell growth is influenced by endogenous growth factors. Transforming growth factor-beta (TGFβ), a multifunctional cytokine expressed in the intestinal epithelium, regulates cell differentiation, proliferation, angiogenesis, and immune response [14]. TGFβ triggers downstream signaling pathways by activating serine/threonine kinase transmembrane receptors type I (TGFβ R1) and type II (TGFβ R2), leading to the phosphorylation of the intracellular mediators SMAD2 and SMAD3 [14,15,16,17]. Phosphorylated SMAD2 or SMAD3 forms a complex with SMAD4, translocating into the nucleus to regulate the transcription of target genes [17]. The TGF β/SMAD2-3-4 pathway is significantly associated with CRC risk in humans [14] and is a potential target for CRC management. For instance, miR-495-3p can negatively regulate the genes encoding TGFβ R1, TGFβ R2, SMAD4, and Bub1, leading to G1 phase arrest in HCT-116 cells [18].

In this study, we undertook the inaugural exploration of the anti-proliferative properties of involucrasin B. This research uniquely contributes to the existing literature by being the first to specifically examine the inhibitory effects of involucrasin B on cell proliferation and its modulation of the TGFβ/SMAD2-3-4 signaling axis in CRC cells. By focusing on this unexplored aspect, we aim to provide a novel perspective on the potential mechanisms underlying the anticancer properties of involucrasin B, offering valuable insights for the development of future therapeutic strategies in CRC treatment.

## 2. Results

### 2.1. Involucrasin B Effectively Inhibits CRC Cell Proliferation

**Figure 1:**
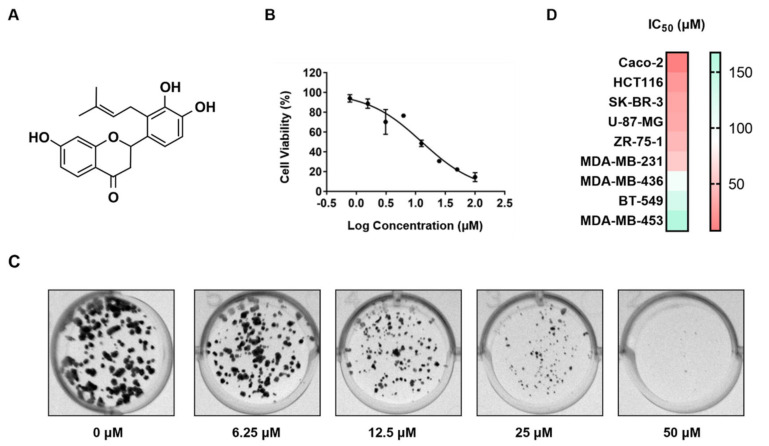
Involucrasin B effectively inhibits Caco−2 cell proliferation. (**A**) Chemical structure of involucrasin B. (**B**) Dose−dependent impact of involucrasin B on 48 h survival rate of Caco−2 cells. (**C**) Cell colony formation following 7−day treatment with involucrasin B (0, 6.25, 12.5, 25, and 50 μM/mL). (**D**) Dose−dependent effect of involucrasin B on viability of various cancer cell lines within 72 h.

Involucrasin B, a white amorphous powder, was isolated from *Shuteria involucrata* by Zhou and Yang with a purity of 100% [13], meeting the requirements of the present experiment. Its molecular formula is C_20_H_20_O_5_, and its structural formula is shown in Figure 1A. To determine the cytotoxicity and optimal dose of involucrasin B in CRC, we treated Caco-2 cells with different concentrations of involucrasin B for 48 and 72 h for SRB screening. The results showed that involucrasin B inhibited the Caco-2 cell growth in a concentration-dependent manner. The IC50 values were determined to be 12.69 µM at 48 h (Figure 1B) and 7.95 μM at 72 h. These findings were further supported by a long-term clone formation experiment, where the Caco-2 cells were continuously treated with involucrasin B for 7 d (Figure 1C). When compared with other cancer cell lines (Figure 1D), involucrasin B showed the most potent anti-proliferative activity against Caco-2, with the IC50 values ranging from 7.9 to 22.7 μM at 72 h. Therefore, this study focused on investigating the anti-proliferative activity and mechanism of action of involucrasin B in Caco-2 cells.

### 2.2. Involucrasin B Induces G1 Phase Arrest of the Cell Cycle and Inhibits Cyclin D1 Expression

**Figure 2:**
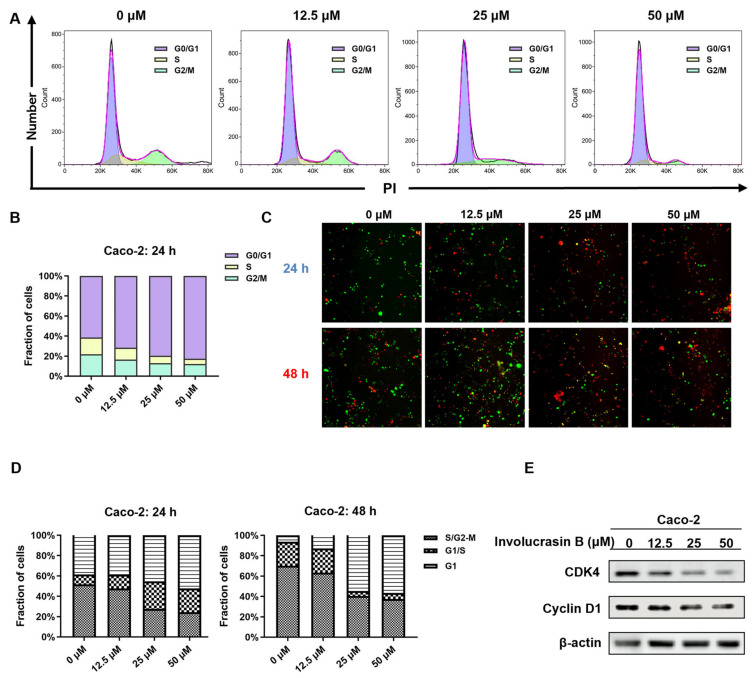
Involucrasin B induces G1 phase cell cycle arrest in Caco-2 cells. (**A**) Caco-2 cells treated with different concentrations of involucrasin B (0, 12.5, 25, and 50 μM/mL) for 48 h, with cell cycle analysis using flow cytometry. (**B**) Statistical chart of A. (**C**) Fluorescence images of FastFUCCI–Caco-2 cells treated with involucrasin B (0, 12.5, 25, and 50 μM/mL) for 24 and 48 h. (**D**) Statistical diagram of C. (**E**) Analysis of cyclin D1 and CDK4 protein expression levels using Western blotting, with β-actin as the loading control. PI, propidium iodide.

In order to ascertain whether the decline in Caco-2 cell proliferation is due to stagnation in the cell cycle, we analyzed the effect of involucrasin B on the cell cycle using PI staining and flow cytometry. Post a 24 h treatment, there was a dose-dependent increase in the proportion of cells in the G0/G1 phase from 61.47% to 82.58% (Figure 2A,B). Concurrently, there was a notable reduction in cells in the S phase and G2. To directly observe the changes induced by involucrasin B in the cell cycle of the Caco-2 cells at the cellular level, we performed a transfection of FastFUCCI into the Caco-2 cells. This ubiquitin-based cell cycle indicator system incorporates mKO2-hCDT1 (red)- and mAG-hGEM (green)-labeled reporter genes into the cells, thereby allowing for continuous monitoring of cell cycle changes via alterations in the fluorescence [19]. The cell populations under red, yellow, and green illumination correspond to cells in the G1, transition from G1 to S, and transition from S to G2-M phases, respectively. Following 24 h of treatment, the proportion of red nuclei increased in a dose-dependent manner from 38.6% to 52.4%, while the number of green nuclei decreased from 51.7% to 24.3%. After the treatment extended to 48 h, the proportion of red nuclei increased from 6.3% to 56.6%, whereas the number of green nuclei decreased from 70.1% to 37.6% (Figure 2C,D). For a more comprehensive understanding of involucrasin B’s impact on the cell cycle distribution, we examined the expression of the G1-phase-related proteins cyclin-dependent kinase 4 (CDK4) and cyclin D1 in vitro. Compared with the control group, the expression of CDK4 and cyclin D1 decreased dose-dependently (Figure 2E).

### 2.3. Involucrasin B Inhibits the CRC Cell Cycle through the TGFβ/SMAD2-3-4 Pathway

**Figure 3:**
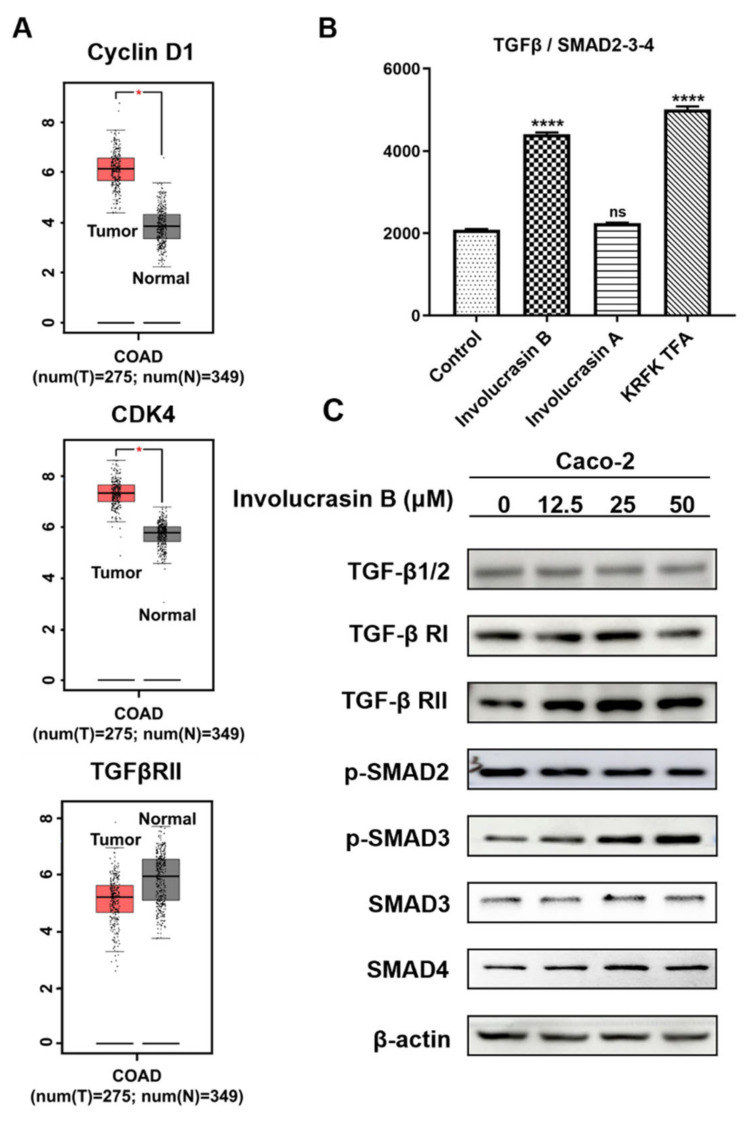
Involucrasin B activates the TGFβ/SMAD2-3-4 pathway to induce cell cycle arrest. (**A**) Analysis of gene expression levels of cyclin D1, CDK4, and TGFRII in colorectal cancer and adjacent normal tissues. Red represents tumor tissue, and gray represents adjacent normal tissue. (**B**) Detection of TGFβ/SMAD2-3-4 expression using double luciferase assay. (**C**) Assessment of TGFβ/SMAD2-3-4 pathway protein expression via Western blotting. (**C**) The protein expression of the TGFβ/SMAD2-3-4 pathway was detected using Western blotting. * *p* < 0.05, **** *p* < 0.0001, compared with control.

Our previous experiments showed that involucrasin B induces Caco-2 cell cycle arrest in the G1 phase. However, the mechanisms underlying cell cycle arrest remain unclear. Bioinformatics analysis confirmed the upregulation of cyclin D1 and CDK4 expression in the CRC cells, while the expression of TGFβ RII was found to be downregulated in the CRC cells (Figure 3A).

Because many studies have shown an abnormal expression of the TGFβ pathway in CRC, we performed a double luciferase reporter gene experiment. Compared with the control vector, TGFβ/SMAD2-3-4 increased significantly after the involucrasin B treatment (Figure 3B), showing similar results to KRFK TFA (TGF-β receptor agonist), while the expression levels of TGFβ/SMAD2-3-4 induced by involucrasin A did not significantly differ from the control vector. To elucidate the effect of involucrasin B on the TGFβ/SMAD2-3-4 pathway in the CRC cells, the Caco-2 cells were treated with different concentrations of involucrasin B (0, 12.5, 25, and 50 μM/mL). After 48 h, the proteins were collected, and the expression levels of related proteins were detected using Western blotting. The involucrasin B treatment significantly enhanced the TGFβ RII expression and SMAD3 phosphorylation dose-dependently (Figure 3C). However, involucrasin B only slightly affected the expression of TGFβ, TGFβ RI, p-SMAD2, SMAD3, and SMAD4. These results suggest that involucrasin B inhibits the proliferation of CRC cells by activating the TGFβ/SMAD2-3-4 pathway through TGFβ RII acting on the Caco-2 cells.

### 2.4. SD-208 Can Attenuate the Proliferation Inhibition of Involucrasin B in Caco-2 Cells

**Figure 4:**
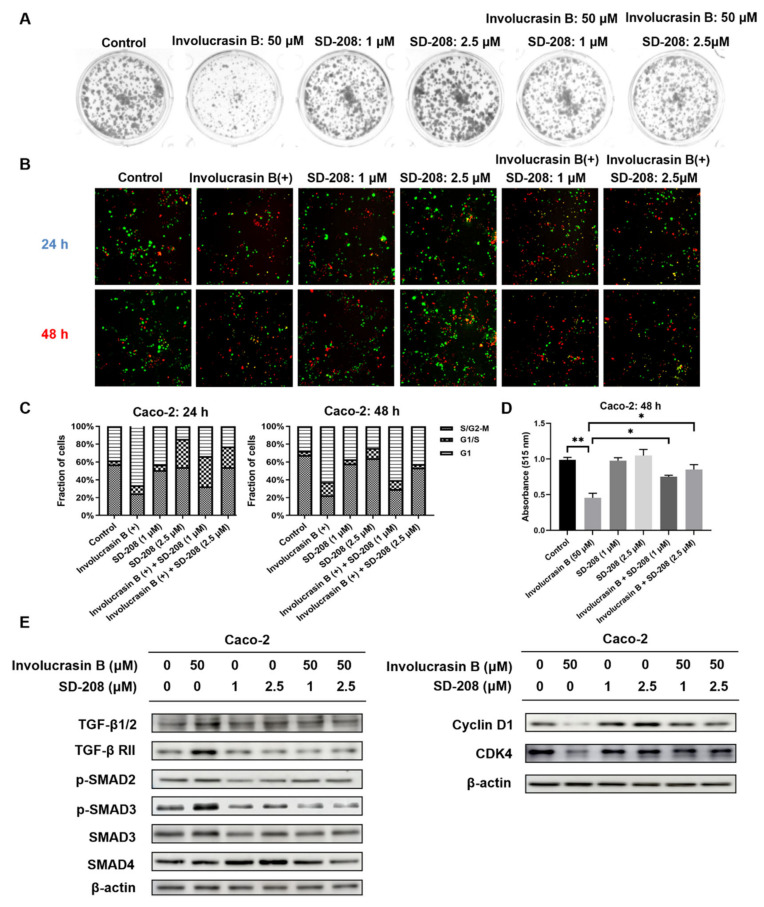
TGFβ receptor inhibitor SD-208 attenuates the anti-proliferative effect of involucrasin B. (**A**) Caco-2 cells treated with SD-208 (1 and 2.5 μM/mL) and involucrasin B (50 μM/mL) for 3 d, followed by fresh medium to promote colony formation for 4 d. (**B**) Fluorescence images of FastFUCCI–Caco-2 cells treated with SD-208 (1 and 2.5 μM/mL) and involucrasin B (50 μM/mL) for 24 and 48 h. The cell populations under red, yellow, and green illumination correspond to cells in the G1, transition from G1 to S, and transition from S to G2-M phases, respectively. (**C**) Statistical chart of B. (**D**) SRB assay to detect cell viability of Caco-2 cells treated with involucrasin B (50 μM/mL) and SD-208 (1 and 2.5 μM/mL) for 48 h. (**E**) Western blotting to assess the expression of related proteins after 48 h of treatment with SD-208 (1 and 2.5 μM/mL) and involucrasin B (50 μM/mL). All SD-208 groups were pretreated for 2 h. All groups containing SD-208 were pretreated for 2 h. * *p* < 0.05, ** *p* < 0.01.

To confirm the mechanism by which involucrasin B exerts its anti-proliferative effect through the regulation of the TGFβ/SMAD2-3-4 signaling pathway, Caco-2 cells treated with involucrasin B were subjected to different concentrations of the TGFβ receptor inhibitor SD-208. The clone formation experiment (Figure 4A; medium replaced after 3 days of administration) demonstrated that SD-208 partially alleviated the inhibitory effect of involucrasin B on the Caco-2 cell proliferation. Fluorescence images of the FastFUCCI–Caco-2 cells were utilized to observe changes in the cell cycle of the CRC cells treated with SD-208. Compared to the control group, the percentage of red G1 phase cells significantly increased from 33.4% to 66.2% at 24 h and from 27.8% to 62.4% at 48 h after treatment with involucrasin B alone (Figure 4B,C). When SD-208 was used alone, Caco-2 cell proliferation was evident, and the proportion of cells in the G1 phase decreased in a concentration-dependent manner. Green Caco-2 cells pretreated with SD-208 showed a dose-dependent increase compared to the cells treated with involucrasin B alone, indicating that more cells completed mitosis through S phase progression to G2/M. SD-208 partially alleviated the G1 phase arrest induced by involucrasin B in the Caco-2 cells. These findings were further confirmed using the SRB experiment with wild-type Caco-2 cells (Figure 4D). Additionally, a Western blotting experiment was conducted to evaluate the impact of SD-208 on the TGFβ/SMAD2-3-4 pathway and G1-phase-related proteins at the protein level. SD-208 downregulated the enhanced expression of TGFβ RII and p-SMAD3 induced by involucrasin B, while upregulating the decreased expression of cyclin D1 and CDK4 caused by involucrasin B. These results suggest that SD-208 reversed the inhibitory effect of involucrasin B on the proliferation of the Caco-2 cells.

### 2.5. SMAD3 Knockdown Weakens the Anti-Proliferative Effect of Involucrasin B

**Figure 5:**
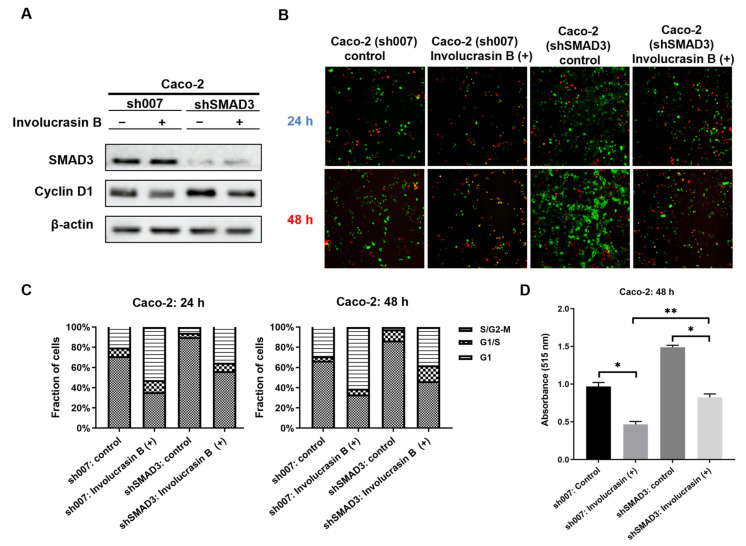
SMAD3 knockdown weakens the anti-proliferative effect of involucrasin B. (**A**) Detection of SMAD3 and CDK4 protein expression levels in Caco-2 cells transduced with sh007 and shSMAD3 using Western blotting. (**B**) Fluorescence images of FastFUCCI–Caco-2 cells transduced with sh007 and shSMAD3 after 24 and 48 h of treatment with involucrasin B (50 μM/mL). The cell populations under red, yellow, and green illumination correspond to cells in the G1, transition from G1 to S, and transition from S to G2-M phases, respectively. (**C**) Statistical chart of B. (**D**) SRB assay to assess the viability of Caco-2 cells treated with involucrasin B (50 μM/mL) for 48 h. * *p* < 0.05, ** *p* < 0.01.

To investigate whether the induction of G1 phase arrest in involucrasin-B-treated Caco-2 cells is mediated through the SMAD2-3-4 pathway, we employed the lentivirus-mediated RNA interference technique to knock down SMAD3 in the wild-type Caco-2 cells and FastFUCCI–Caco-2 cells. The expression of total SMAD3 protein significantly decreased in the cells transduced with shSMAD3; however, there was no significant difference observed after the treatment with involucrasin B (Figure 5A). The knockdown of SMAD3 attenuated the decrease in the CDK4 protein levels induced by the involucrasin B treatment, but a complete reversal was not observed, suggesting that involucrasin B induces a decrease in CDK4 expression through alternative pathways. The proliferation rate of the FastFUCCI–Caco-2 cells transduced with shSMAD3 was significantly higher than that of the cells transduced with sh007 (Figure 5B,C). Even after the treatment with involucrasin B, both cell types exhibited significant proliferation inhibition; however, the proportion of G1 phase cells in the shSMAD3-transduced cells was higher compared to in the sh007-transduced cells, indicating that blocking the SMAD3 pathway partially reverses the G1 phase cell cycle arrest induced by involucrasin B. These conclusions were further supported by the SRB experiment conducted with the wild-type Caco-2 cells (Figure 5D). These findings suggest that while SMAD3 phosphorylation is activated by involucrasin B treatment, its subsequent effect on G1 phase cell cycle arrest is not solely dependent on the SMAD2-3-4 pathway.

## 3. Discussion

The mammalian cell cycle encompasses G1, S (DNA synthesis), G2, and M (mitosis) [20,21,22]. Cyclin-dependent protein kinases (CDKs) regulate the cell transformation. Mitogens, hormones, and growth factors stimulate D-type cyclins to activate CDK4 and CDK6, initiating Rb protein family phosphorylation in the early G1 phase [20,22,23]. This inactivation of Rb leads to E2F transcription factor release, promoting the transcription of E2F response genes for cell cycle progression [3,20,22,24]. Later in the G1 phase, the CDK2/cyclin E complex hyperphosphorylates Rb, crossing the G1/S checkpoint and initiating the S phase. CDK2, along with cyclin A, is crucial during the S phase, while the CDK1/cyclin B complex controls mitosis completion [20,21,22]. These processes collectively regulate the orderly progression of the cell cycle.

In the past two decades, CDK4 and CDK6 have emerged as crucial drivers of cell cycle dysregulation in carcinogenesis [3]. The overexpression of CDK4/6 due to genomic alterations is common in most human cancers [21]. Cyclin D1, an important regulator of the G1/S cell cycle checkpoint, forms a complex with CDK4/6, leading to uncontrolled cell proliferation [20,22]. Inhibiting the cyclin D1 and CDK4/6 complex can induce G1 phase arrest in cancer cells. CDK4/6 inhibitors have shown clinical efficacy against advanced breast cancer and lymphomas [20,21]. This study confirmed G1 phase arrest in involucrasin-B-treated Caco-2 cells using flow cytometry and FastFUCCI assay, along with the downregulation of the cyclin D1 and CDK4 protein levels, as revealed using Western blot analysis (Figure 2E).

Involucrasins A and B are two pairs of enantiomers of flavonoids isolated from *Shuteria involucrata*. The extraction yields of involucrasin A and involucrasin B were found to be 0.64% and 0.56%, respectively [13]. Both compounds exhibited significant inhibitory effects on CRC cells, although their specific inhibitory mechanisms differed. Involucrasin A induced cell death in CRC cells by modulating the Akt/MDM2/P53 pathway [8], while involucrasin B, as investigated in this study, suppressed CRC cell proliferation by regulating the TGFβ/SMAD2-3-4 pathway. Comparative and repetitive analysis of the nuclear magnetic resonance spectra revealed that the ~1H and ~(13) C NMR spectra of involucrasin B were highly similar to those of involucrasin A, except for the absence of the methoxy signal in involucrasin B [13]. Further experimental research is required to determine whether the differential activity between the two compounds is due to variations in their functional groups.

This study primarily focused on investigating the pharmacological mechanism of involucrasin B. As a tool compound for the TGFβ/SMAD2-3-4 pathway, SD-208 was employed. Upon the addition of SD-208, we did observe partial rescue of the cell cycle alterations, clonogenic formation, and SRB cell numbers, as indicated by the FastFUCCI–Caco-2 system. The Western blot analysis also revealed partial rescue of the CDK4 and cyclin D1 expression. Although we observed an increase in the SMAD4 protein expression when SD-208 was used alone, we did not intend to extensively study the other pharmacological mechanisms of SD-208 in this research. Similarly, after the shSMAD3 treatment in the Caco-2 cells, we observed similar changes. Therefore, we believe that the alterations in the TGFβ/SMAD2-3-4 pathway induced by involucrasin B in the Caco-2 cells are part of the mechanism responsible for G1 phase cell cycle arrest. This study primarily revolves around the impact of involucrasin B on the G1 phase arrest of Caco-2 cells through the activation of the TGFβ/SMAD2-3-4 pathway. However, it is acknowledged that G1 phase arrest may not be exclusively mediated through this pathway. For instance, previous research has demonstrated that p53 induces G1 cell cycle arrest via the upregulation of p21^cip1/waf1^ in response to cellular stresses [25,26]. Whether involucrasin B influences the functionality of p53 remains to be elucidated. Further investigations could delve into the potential impact of involucrasin B on the p53 pathway and its involvement in the G1 phase arrest of Caco-2 cells. This endeavor would contribute to a more comprehensive understanding of the mechanistic role of involucrasin B in cell cycle regulation, thus providing insights for the treatment and prevention of relevant diseases.

One limitation of this study is the predominant focus on a single cell line (Caco-2 cells) due to the insufficient availability of involucrasin B, which might constrain the generalizability of the results. Therefore, in order to enhance the generalizability of the findings, it would be beneficial to validate these observations in other CRC cell lines (such as HCT-116, for instance). Conducting validation across different cell lines would allow for a more comprehensive understanding of the impact of involucrasin B, bolstering the reliability of the study conclusions and providing a more robust basis for potential applications in the field of CRC therapy. Consequently, in order to attain a more thorough comprehension of the effect of involucrasin B on CRC, future research endeavors may consider expanding the scope of the sample to encompass a variety of CRC cell lines.

Another limitation of this study is the insufficient quantity of involucrasin B available for in vivo animal experiments. This constraint implies a hindrance to the progression of the clinical translation of the potential therapeutic effects of involucrasin B. In order to further evaluate the efficacy and safety of involucrasin B in in vivo models, future research will require an adequate supply of involucrasin B for animal experiments. These experiments will contribute to assessing the impact of involucrasin B on tumor development, growth, and metastasis, as well as its potential toxic side effects. This information will be crucial in supporting the application of involucrasin B in clinical trials and the treatment of patients.

In terms of the clinical prospects, involucrasin B may serve as an effective treatment option, particularly for colorectal cancer patients with excessive CDK4 activation and aberrant TGFβ signaling pathways. By inhibiting CDK4 activity, involucrasin B can impede the proliferation and division of cancer cells, thereby restraining tumor progression.

In conclusion, our study illustrates that involucrasin B targets the TGFβ RII receptors on the cell membranes to affect Caco-2 cells. This interaction stimulates SMAD3 phosphorylation and activates the SMAD2-3-4 signaling pathway, resulting in the inhibition of cyclin D1/CDK4 expression and leading to G1 cell cycle arrest in colon cancer cells. These findings provide important insights into the molecular mechanisms underlying the anti-proliferative properties of involucrasin B and its potential as a therapeutic agent for colon cancer.

## 4. Methods and Materials

### 4.1. Reagents

The involucrasin B was a white amorphous powder isolated from *Shuteria involucrata* by Zhou and Yang [13]. The involucrasin B was dissolved in dimethyl sulfoxide (Thermo Fisher Scientific) and preserved at –40 °C. The sulforhodamine B (SRB), trichloroacetic acid (TCA), puromycin, Tris base, and crystal violet were purchased from MilliporeSigma (Burlington, MA, USA). The minimum essential medium (MEM), RPMI-1640, Dulbecco’s modified Eagle medium (DMEM), and fetal bovine serum (FBS) were purchased from Gibco (Thermo Fisher Scientific, Waltham, MA, USA). The TGFβ/SMAD inhibitor SD-208 was purchased from Beyotime Institute of Biotechnology (Shanghai, China). The TGF-β receptor agonist KRFK TFA was purchased from MedChemExpress (Monmouth Junction, NJ, USA).

### 4.2. Cell Lines and Culture

The CRC (Caco-2 and HCT-116), breast cancer (SK-Br-3, ZR751, MDA-MB-231, MDA-MB-436, BT-549, and MDA-MB-453), and glioblastoma (U-87-MG) cell lines were obtained from the American Type Culture Collection. All media were supplemented with 10% FBS and 1% antibiotics (100 U/mL penicillin and 0.1 mg/mL streptomycin). All cells were cultured in a humidified incubator containing 5% CO_2_ at 37 °C.

### 4.3. Cell Viability Assay

The cells were plated in a 96-well plate at a concentration of 5 × 10^3^ cells per well and treated with varying concentrations of involucrasin B (0–200 μM) in fresh culture media. The plate was then incubated in a 5% CO_2_ environment at 37 °C for 48 or 72 h. The cell viability was assessed using an SRB colorimetric assay, where the cells were fixed and dyed and their absorbance was measured at 515 nm using a SpectraMax absorbance microplate reader.

### 4.4. Cell Cycle Analysis

The cell cycle was analyzed using flow cytometry. The Caco-2 cells were seeded in 6-well plates overnight at a density of 4 × 10^5^ cells/well. The cells were then treated with different concentrations of involucrasin B (0, 12.5, 25, and 50 μM) in MEM and incubated at 5% CO_2_ and 37 °C for 24 h. After washing with 1× PBS, the cells were digested with trypsin, centrifuged, and fixed using 70% ethanol at –20 °C for 2 h. Then, the cells were stained with PI/RNase A (Nanjing KeyGen Biotech Co., Ltd. Najing, China) for 30 min at room temperature. Finally, the cells were washed, centrifuged, and suspended in 1× PBS. The cell fluorescence was detected using a FACSAria II flow cytometry system (BD Biosciences, San Jose, CA, USA), and the data were analyzed using FlowJo 10.8.1.

### 4.5. Colony Formation Assay

To study the effect of involucrasin B on the colony-forming ability of Caco-2, different cell seeding densities were used in 12-well plates and 24-well plates. The cells were treated with varying concentrations of involucrasin B for different durations, and in the case of the 24-well plates, a combination treatment with involucrasin B and SD-208 was applied. Following incubation, the cells were washed and stained, and colony images were documented using Molecular Imager Gel Doc^TM^ XR+ (Bio-Rad, Hercules, CA, USA).

### 4.6. Dual Luciferase Assay

HEK293T cells were seeded onto a 96-well plate at a density of 10 × 10^4^ cells per well. The cells were then transfected with the target plasmid (TGF β/SMAD2-3-4, 120 ng/well) using FuGENE HD. The plate was incubated overnight at 37 °C in a 5% CO_2_ incubator. On the second day, involucrasin A, involucrasin B, and KRFK TFA were added to the 96-well plate at final concentrations of 100 μM, 50 μM, and 50 μM, respectively. The plate was incubated overnight at 37 °C in a 5% CO_2_ incubator. On the third day, the luciferase activity was determined using the luminescence-FLuc protocol with VICTOR 2, according to the manufacturer’s instructions. The NLuc luminescence was normalized to the FLuc luminescence, and the ratio (relative activity) was plotted against pathways/transcription factors and analyzed. 

### 4.7. Western Blot Analysis

The expression levels of the proteins extracted from the Caco-2 cells were detected using Western blotting. The Caco-2 cells were collected after treatment with involucrasin B (0–50 μM) and SD-208 (0–2.5 μM) for 48 h. RIPA cleavage buffer (Beijing Solarbio Science & Technology Co., Ltd. Beijing, China) containing a protease inhibitor (Roche Diagnostics, Risch-Rotkreuz, Switzerland) and a phosphatase inhibitor (Roche Diagnostics, Risch-Rotkreuz, Switzerland) were used to prepare the whole cell lysate.

The BCA Protein Assay Kit (Beyotime Institute of Biotechnology, Shanghai, China) was employed to determine the protein sample concentrations. Protein supernatant was mixed with upper sample buffer and separated onto 10% SDS-PAGE gel. The resulting proteins were transferred to PVDC membranes, blocked in 5% milk or 5% BSA for 1 h, and then incubated with the primary antibody overnight at 4 °C. After washing with TBST buffer, the membranes were incubated with peroxidase secondary antibodies for 1 h at room temperature. Subsequently, the membranes were washed thrice with TBST and then with once with TBS before the visualization of the relative protein expression using either SuperSignal West Pico PLUS Chemiluminescent Substrate (Thermo Fisher Scientific, Inc., Waltham, MA, USA) or UltraSignal hypersensitive ECL chemiluminescence substrate (4A Biotech) and quantification with an Amersham 800/600 imager (Cytiva, Marlborough, MA, USA) and imaging software. The antibodies were diluted according to the provided instructions. The primary antibody cyclin D1 was obtained from Cell Signaling Technology. The CDK4, TGFβ, TGFβ RI, TGFβ RII, SMAD3, phospho-SMAD3, phospho-SMAD2, and SMAD4 were obtained from Beyotime Institute of Biotechnology. The anti-rabbit and anti-mouse secondary antibodies were obtained from Jackson ImmunoResearch Laboratories, Inc (West Grove, PA, USA). 

### 4.8. Cell Transfection

Short hairpin RNA (shRNA; pLVX-U6-SMAD3 (human) shRNA3-PGK-EGFP-E2A-Puro) of SMAD3 with structural activity was successfully expressed. The SMAD3 shRNA (human) plasmid was purchased from Miaoling Biology, Inc. (Wuhan, China). An empty plasmid was used as a control. The shRNA control plasmid (sh007) was purchased from MilliporeSigma (insert sequence: 5′-CCG GCG CTG AGT ACT TCG AAA TGT CCT CGA GGA CAT TTC GAA GTA CTC AGC GTT TTT-3′). Lentivirus was generated by transducing the HEK293T cells with the specified construct, psPAX2 (Addgene), and pMD2.G (Addgene) in Opti-MEM (Gibco; Thermo Fisher Scientific, Waltham, MA, USA) with FuGENE HD (Promega Corporation, Madison, WI, USA). After 48 h, the collected culture medium was filtered, frozen, and added to the Caco-2 cells. Following a 24 h incubation with lentivirus, puromycin (Thermo Fisher Scientific, Waltham, MA, USA) was employed for the cell screening over 3 days. The puromycin concentration range used for selection and maintenance was 1–10 μg/mL.

### 4.9. FastFUCCI Assay

To visualize the cycle changes in the nucleus, we used the FastFUCCI reporting system for the cell cycle analysis in the Caco-2 cells. The pBOB-EF1-FastFUCCI-Puro plasmid was purchased from Addgene. The FastFUCCI reporting system has been used to clone Gminin-MAg and CDT-mKO2 fusion genes into the pTP6 system to achieve stable transgenic expression based on puromycin or neomycin resistance [19]. The obtained vector was co-transfected into Caco-2 using GenJet^TM^ Plus according to the manufacturer’s instructions. After the puromycin selection, a single Caco-2 colony containing green and red fluorescent cells was selected for further analysis. The subcloned FastFUCCI–Caco-2 cells were cultured in the presence of puromycin to ensure constant and homogeneous expression of the reporter gene. The fluorescence data were collected using an IN Cell Analyzer 6000 laser confocal imaging system, and the images were analyzed using Image J2.

### 4.10. Bioinformatics Analysis

The CCND1 (cyclin D1), CDK4, and TGFBR2 (TGFβ RII) in CRC and normal tissues were analyzed by http://gepia.cancer-pku.cn/(accessed on 29 September 2023).

### 4.11. Statistical Analysis

GraphPad Prism 8.0 was used for the statistical analysis. The data are expressed as mean ± SD of at least three independent repetitions. The statistical difference between the two groups was evaluated using Student’s *t*-test. For comparisons involving three or more groups, a one-way analysis of variance (ANOVA) followed by Tukey’s multiple-comparison test was utilized. *p* < 0.05 was considered statistically significant.

## Data Availability

The datasets used and/or analyzed during the current study are available from the corresponding author on reasonable request.
